# Confounding Factors in the Association Between Glucagon-Like Peptide-1 Receptor Agonist Use and Retained Gastric Contents in Asymptomatic Patients Undergoing Upper Gastrointestinal Endoscopy: A Retrospective Study

**DOI:** 10.7759/cureus.69152

**Published:** 2024-09-11

**Authors:** Thomas A Elimihele, Anjali M Mangrola, Oluwatobi Oshomoji, Nateshia B Wilson, Ikenna Nnamani, Bryan Ashong, Sunteasja Billings, Daniel K Getu, Sachin Kumar, Benedict Maliakkal

**Affiliations:** 1 Department of Internal Medicine, Meharry Medical College, Nashville, USA; 2 Department of Population Health/Analytics, Nashville General Hospital, Nashville, USA; 3 Department of Internal Medicine, Spartan Health Sciences University School of Medicine, Vieux Fort, LCA; 4 Department of Gastroenterology and Hepatology, Nashville General Hospital, Nashville, USA

**Keywords:** anticholinergics, aspiration of gastric contents, aspiration of gastric contents, confounding factors, : delayed gastric emptying, esophagogastroduodenoscopy (egd), glucagon-like peptide-1 receptor agonist, opiates, semaglutide, colonoscopy

## Abstract

Introduction

The use of glucagon-like peptide-1 receptor agonists (GLP-1 RA) has gained acceptance in managing diabetic and non-diabetic patients, thanks to its benefits in treating obesity and improving cardiovascular outcomes. The potential ability of GLP-1 RA to cause retained gastric contents (RGC) which can lead to aspiration has led to the recommendation to withhold GLP-1 RA for at least one week prior to elective surgeries and procedures, including upper endoscopies and colonoscopies. However, many co-medications and conditions associated can contribute to delayed gastric emptying (DGE) and these need to be controlled to establish a clear association and incidence, which has been largely missing in most studies and reports. Our aim was to assess clinically significant delayed gastric emptying (CSDGE) related to GLP-1 RA in a “real world” situation by controlling for some common confounding factors.

Method

We carried out an eight-year retrospective single-center study to assess the relationship between CSDGE and the use of GLP-1 RA among asymptomatic patients undergoing upper endoscopies while controlling for common confounding factors.

Result

Out of the 3415 patients who had esophagogastroduodenoscopy (EGD) with or without colonoscopy (Ew/woC) during the eight-year period, 129 eligible patients were found to have CSDGE. The use of GLP-1 RA was associated with the lowest percentage frequency distribution of CSDGE, at 2%, and opiate accounted for more percentage frequency distribution, at 35%. The odds ratio for patients on GLP-1 RA who developed CSDGE was 2.5 (95% CI 0.75-8.29). All patients who had CSDGE while on GLP-1 RA were also on other medications or had conditions associated with DGE.

Conclusion

Our result confirmed the possible effect of confounding factors in the association between CSDGE and GLP-1 RA among asymptomatic patients undergoing Ew/woC. While the need to ensure patients' safety cannot be overemphasized, the proper approach currently favors assessing patients on a case-by-case basis while we await the results of large prospective controlled studies.

## Introduction

Since the launch of exenatide, the first glucagon-like peptide-1 (GLP-1) receptor agonist (GLP-1 RA) approved by the United States Food and Drug Administration (FDA) for the management of type 2 diabetes mellitus (T2DM) in the United States in 2005 [1], several other medications in this class have been approved not only for T2DM but for other clinical indications. Some approved medications in this class include exenatide, liraglutide, dulaglutide, semaglutide, and tirzepatide by the FDA.

GLP-1 RA works by mimicking the actions of GLP-1, an endogenous incretin hormone, binding to GLP-1 receptors in the pancreas to stimulate insulin release in a glucose-dependent manner [2]. Due to the expression of GLP-1 receptors in other cells, including the hypothalamus, gut, stomach mucosa, heart, lungs, kidneys, and immune system [2, 3], these agents have been shown to modulate other effects such as delaying gastric emptying, inducing early satiety, and blocking the abnormal release of glucagon in the post-meal period [4,5].

GLP-1 agonists have become useful in many clinical conditions besides T2DM for which it was initially approved, including in patients with obesity (body mass index (BMI) of 30 and above) and patients with BMI of 27 and above with at least one weight-related complication [6]. Some formulations like semaglutide, liraglutide, and dulaglutide even have the additional benefits of reducing rates of acute myocardial infarction, stroke, and cardiovascular death in patients with T2DM [7].

Despite all their benefits, GLP-1 RAs have been associated with side effects that may limit their use. Apart from the previously well-documented disturbances in gastrointestinal (GI) functions manifesting as symptoms of nausea, vomiting, and bloating, recent reports have started to link these medications with increased incidence of gastroparesis and delayed gastric emptying (DGE) [8-21]. However, there have also been reports of no significant difference in gastric emptying with some GLP-1 RA and placebo [22, 23].

Adequate gastric emptying is an important requirement for successful endoscopy procedures, especially upper gastrointestinal (GI) endoscopies due to the need for proper visualization of the stomach and, most importantly, to increase the safety of the procedure by reducing the risk of aspiration of retained gastric contents (RGC) in sedated patients with limited abilities in managing their airways. These standards for endoscopy and concern for GLP-1 RA-associated DGE prompted the American Society of Anesthesiologists (ASA) to create a consensus-based guideline on perioperative management of patients on GLP-1 RAs, with recommendations for withholding these medications prior to carrying out any elective surgery or procedures including endoscopies [24]. Yet, it is unclear if GLP-1 RA use alone can be posited as the culprit of DGE given that there are multiple agents and disease conditions associated with DGE [25, 26]. Top among these are known causes of gastroparesis such as long-standing diabetes mellitus, Parkinson's disease, acute viral illness as well as several other medication classes including anticholinergics, tricyclic antidepressant, and opioids [25, 26]. Patients with blood sugars above 200 mg/dL may also have significant impairment of gastric emptying or short-term reversible gastroparesis [26]. These factors can complicate or confound the findings of DGE in patients who are on a GLP-1 RA.

To this end, we aim to carry out a preliminary analysis of data in a large urban hospital to study the association between GLP-1 RA use and clinically significant delayed gastric emptying (CSDGE) among asymptomatic patients undergoing esophagogastroduodenoscopy (EGD) while accounting for confounding factors such as the use of other medications capable of causing DGE and common comorbidities that have been linked with DGE and gastroparesis.

## Materials and methods

This study was approved by the institutional review board of Meharry Medical College and Nashville General Hospital (NGH) on December 12, 2023, with the approval number FWA00003675.

We carried out a retrospective chart review of all patients who had EGDs with or without colonoscopy (Ew/woC) at NGH from January 2015 to December 2023. We chose 2015 because most long-acting GLP-1 RAs currently in clinical practice in the United States (US) were approved after this period [27]. We included all patients who had prepared for Ew/woC with an overnight fast after midnight on the day of their EGD and excluded those who had emergent EGDs due to foreign body ingestion, upper gastrointestinal bleeding (UGIB) or any other emergent reason that prevented them from fasting. We also excluded patients with known diagnoses of gastroparesis, gastric outlet obstruction, and those on gastrointestinal (GI) motility agents such as metoclopramide, domperidone, and erythromycin for symptomatic management of symptoms of delayed gastric movement. We selected patients with EGD findings of retained food, retained partially digested food, phytobezoars, and other findings suggestive of DGE. Patients with any of the above findings or its combination were designated as patients with CSDGE. For every patient with CSDGE, we recorded their demographic information excluding their personal protected information, basal metabolic index (BMI), use of GLP1 RA, duration of use of GLP1 RA, use of medications with anticholinergic properties that have been linked with significant or intermediate risk of delayed gastric emptying or gastroparesis as shown in Table 1, and use of opioids at the time of (Ew/woC). We also recorded those with a diagnosis of diabetes mellitus, hemoglobin A1c two weeks before and after endoscopy, and their blood glucose level on the morning of the procedure. To ensure that patients were actually on GLP-1 RA and the other medications, only patients who were actively filling these medications before and after the procedure or those who had inpatient records confirming the administration of these medications at the time of Ew/woC were included. In addition, we recorded all patients who had Ew/woC within the period of January 2015 and December 2023 who were on GLP1 RA during the time of their EGD irrespective of the presence or absence of DGE. Finally, we assessed the presence of CSDGE in patients on GLP1 RA who had both EGD and colonoscopy on the same day.

**Table 1:** A list of anticholinergic drugs among patients with CSDGE CSDGE= clinically significant delayed gastric emptying

List of Anticholinergic drugs
Dicyclomine
Oxybutynin
Hyoscine
Glycopyrrolate
Hyoscyamine
Benztropine
Tolterodine
Fesoterodine
Tricyclic antidepressants
Antipsychotics

Using mostly simple descriptive statistics, we created tables and charts to analyze the relationships between CSDGE on EGD and the associated use of GLP1 RA, as well as the association with other agents and conditions associated with DGE. We also calculated odds ratios (Table 2) to determine if there was any statistically significant association between the development of CSDGE and the use of GLP-1 RA. We used the 95% confidence interval as a statistical test of significance in the development of CSDGE among asymptomatic patients on GLP-1 RA.

**Table 2:** The 2x2 table used in calculating the odds ratio of patients with CSDGE on GLP-1 RA GLP-1 RA- Glucagon-like peptide-1 receptor agonist, CSDGE- clinically significant delayed gastric emptying,  DGE: delayed gastric emptying

	GLP-1 RA	No GLP-1 RA	Total
DGE	3	126	129
No DGE	31	3255	3286
Total	34	3381	3415

## Results

There were 3415 patients who had elective Ew/woC for various indications from January 2015 - December 2023; of these patients, 1609 were female (47%) and 1806 were male (53%). 129 of these patients had CSDGE (3.78%) while 3286 patients had no CSDGE. A detailed breakdown of the demographic information of the patients with CSDGE is shown in Table 3. 

**Table 3:** Gender and racial distribution of patients who had EGD with or without colonoscopy from 2015-2023 n=number, EGD=Esophagogastroduodenoscopy

Gender and racial distribution	n (%)
Male	1806 (53%)
Female	1609 (47%)
Whites	1287 (38%)
Blacks	1446 (42%)
Others/unknown	682 (20%)

**Figure 1:**
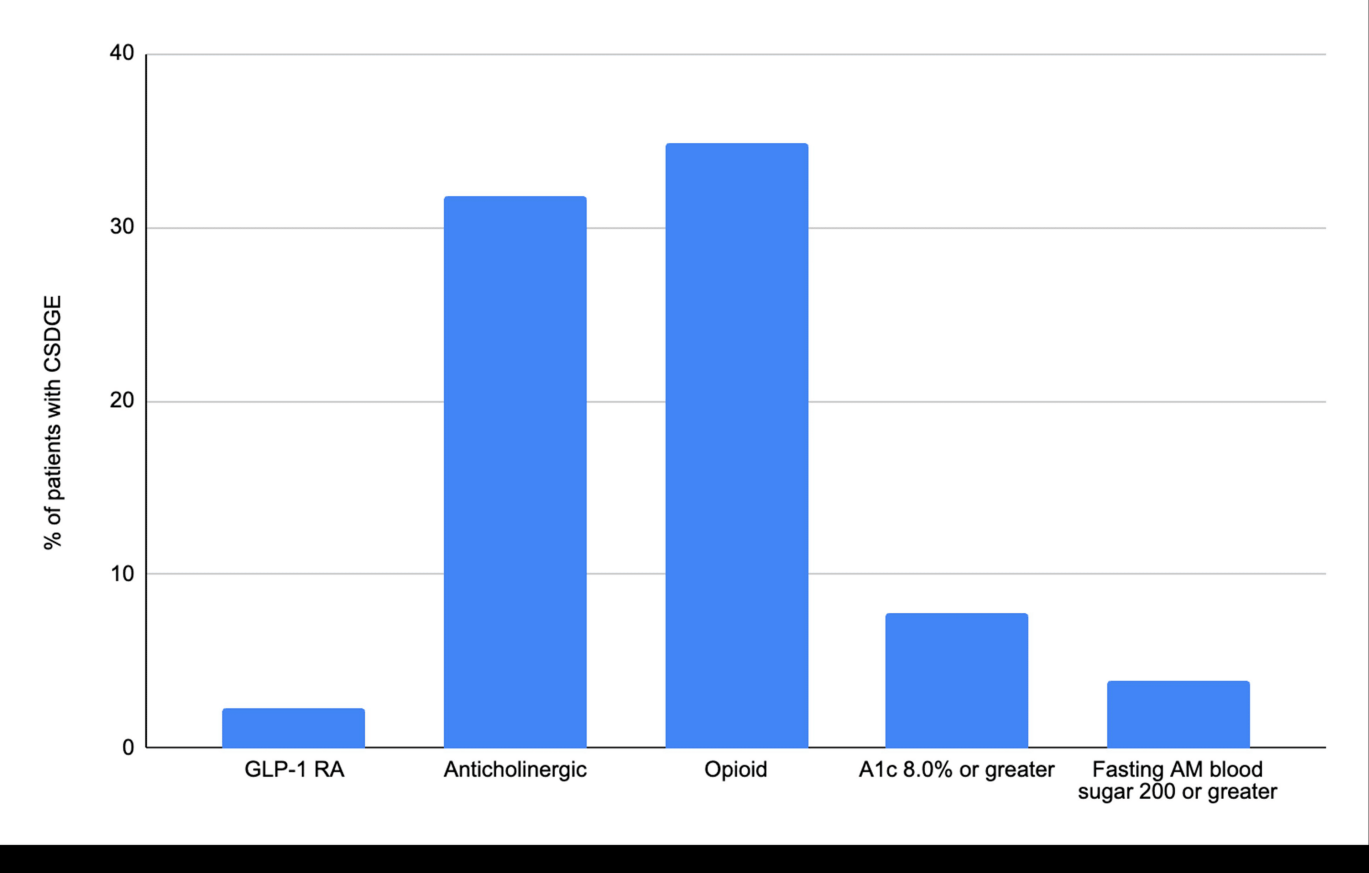
Bar chart showing the percentage frequency distribution of CSDGE among patients on GLP-1 RA and other agents or with clinical conditions/states associated with DGE/gastroparesis GLP-1 RA=Glucagon-like peptide-1 receptor agonist, AM= ante meridiem, CSDGE=clinically significant delayed gastric emptying, DGE=delayed gastric emptying

**Figure 2:**
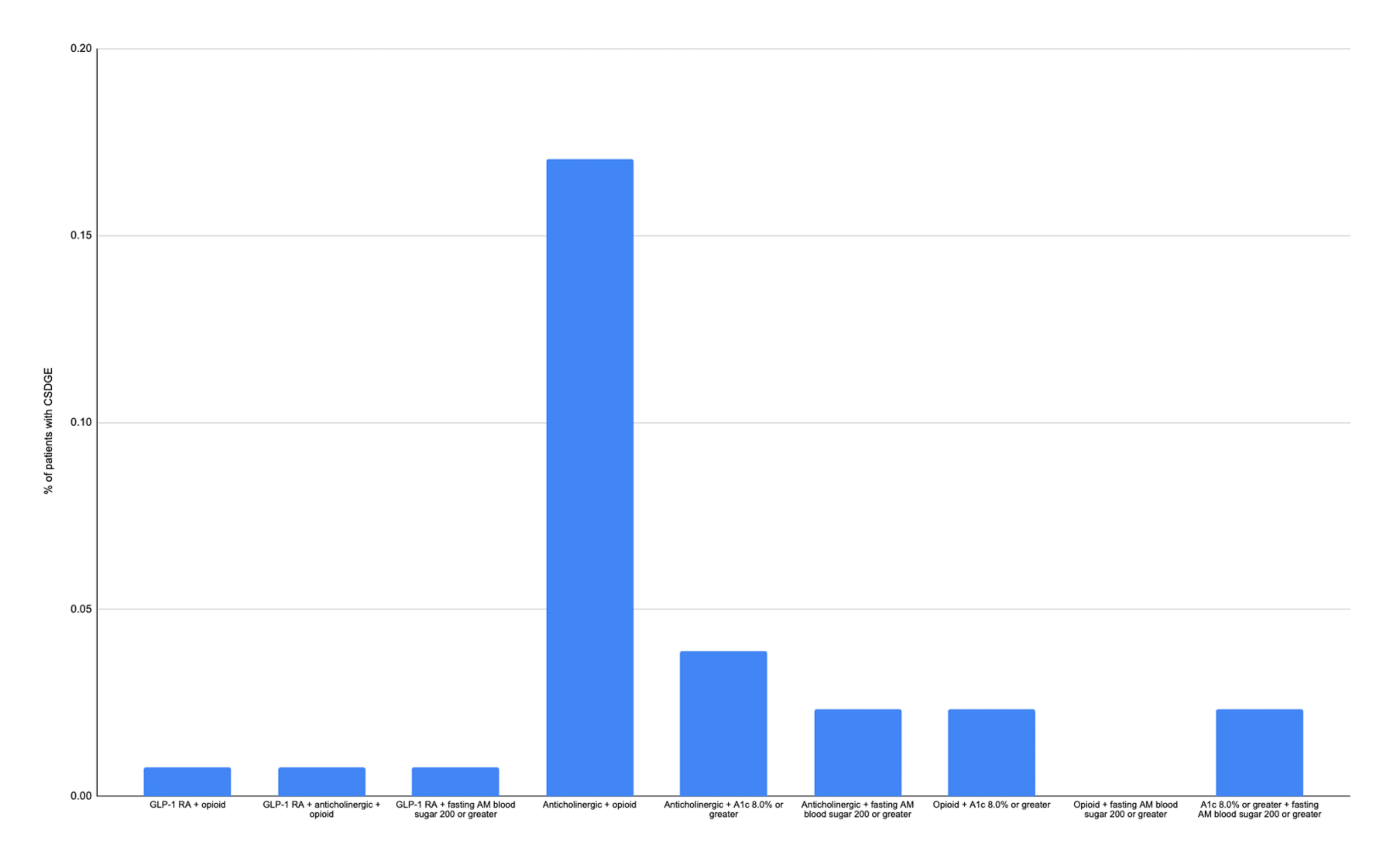
Bar chart showing the percentage frequency distribution of CSDGE among patients on two or more agents or with clinical conditions/states associated with DGE/gastroparesis GLP-1 RA=Glucagon-like peptide-1 receptor agonist, CSDGE=clinically significant delayed gastric emptying, AM= ante meridiem, DGE= delayed gastric emptying

The average age of patients with CSDGE was 50.8, with a standard deviation of 12.9. Thirty-four patients with CSDGE were on GLP-1 RA at the time of Ew/woC, and as shown in Table 4 and Figure 1, only 2% had CSDGE. The odds ratio for patients on GLP-1 RA who developed CSDGE was 2.5 (95% CI 0.75-8.29). Patients on opiates had the highest percentage frequency distribution of CSDGE at 35%. A detailed representation of the percentage frequency distributions of the various agents and clinical conditions/states or a combination of agents and clinical conditions/states among patients with CSDGE is shown in Table 4 and Figures 1, 2.

**Table 4:** Percentage frequency distribution of patients with CSDGE on agents or in clinical conditions/states associated with DGE/gastroparesis GLP-1 RA = glucagon-like peptide-1 receptor agonist, BG=blood glucose, AM=ante meridiem, DM= Diabetes Mellitus; CSDGE: clinically significant delayed gastric emptying; DGE: delayed gastric emptying

Agents and clinical conditions/states	Percentage distribution n (%)
GLP-1 RA use	3 (2)
Opioid use	45 (35)
Anticholinergic use	41 (32)
BG >/= 200 in AM	5 (4)
Hemoglobin A1c >/= 8	10 (8)
Long standing DM	36 (28)

We also looked at patients who had EGD and colonoscopy on the same day while on GLP-1 RA. We reviewed patients from 2015 - 2023 as already described above, and during this period 94 patients were prescribed GLP-1 RA. 24 of these patients had EGD and colonoscopy on the same day, but only 22 were already on GLP-1 RA at the time of the procedure, while the other two started after the procedure. Of the 22 who were taking GLP-1 RA, only one had EGD findings of DGE.

## Discussion

In our study, among the agents and conditions associated with CSDGE on EGD, patients on GLP-1 RA accounted for only three (2%) of the 129 patients with CSDGE. As shown in Figure 1, the percentage frequency distribution of CSDGE was higher in patients with other variables. For example, 35% of patients with CSDGE were on opiates. In Figure 2, among the three patients who had CSDGE while on GLP-1 RA, one of the patients was also on an opiate at the time of the EGD, the second patient was on an opiate and an anticholinergic, and the last patient had a blood sugar of 302 on the morning of the EGD. Our study showed a non-statistically significant increased odds of being on a GLP-1 RA when EGD shows CSDGE (2.5 (95% CI 0.75-8.29)), like some prior studies [22, 23]. This is at variance with the results of many other previous studies and case reports [8-21]; it is important to point out that even with a statistically significant association, the clinical relevance of such an association could still be questioned, given the possibility of effects of confounding factors as seen above. Our study aimed to elucidate the extent of the problem of CSDGE by endoscopy in a "real-world" setting among asymptomatic patients and the medications and other factors associated with it.

There is no doubt that the consequences of aspirating RGC can be severe, a concern shared by both the ASA and the American Gastroenterological Association (AGA) in their guidelines [24, 28], even though the AGA differs on the extent to which the ASA guidelines should be used in patients needing elective endoscopic procedures [28]. The evidence linking GLP-1 RA use to DGE among asymptomatic patients undergoing elective endoscopies is variable and inconsistent. 

Some of the reports that showed this association were case reports [9-11]. Though these reports showed a temporal trend between the start of a GLP-1 RA and the onset of DGE or gastroparesis, the patients, in some instances, had poorly controlled diabetes. The duration of their illness was not reported [9,10], which raises the question of possible confounders, and diabetes mellitus has been strongly linked with gastroparesis [25, 26]. However, in a case report by Klein and Kobai, which was one of the references cited in ASA guidelines, the patient reported was not a diabetic and was not on any medication associated with gastroparesis [11].

Studies that have linked GLP-1 RA with DGE employed several methods to assess gastric emptying, including the use of a C-13 breath test, paracetamol absorption test, and EGD. However, most of these studies were carried out in diabetics [12-19], and even though efforts were made by excluding patients with diagnosis of gastroparesis and diabetic neuropathy [17-19], there could have been a significant number of patients with non-diagnosed or subclinical DGE that were missed and may have been minimally exacerbated by concomitant GLP-1 RA administration. In addition, there was no mention of effort to assess for acute hyperglycemia, during the time of the procedure, which has been linked with DGE [25, 26]. Some of the studies made efforts to control opiate use [18], and some others reported holding medications that could interfere with results [19-21]. A commonality among these studies and reports was the inability to completely account for major confounders of DGE among patients on GLP-1 RA. This is unsurprising given the many agents and conditions associated with DGE and/or gastroparesis [25, 26].

Our study also assessed the possible effect of pre-procedural bowel preparation for colonoscopy on CSDGE among patients taking a GLP-1 RA. The result, which did not include a significance test, showed that out of 22 patients who were on GLP-1 RA at the time of their EGD and colonoscopy, RGC or CSDGE was on seen in one, and this patient, who was also among the three patients reported earlier, was on an anticholinergic and an opiate at the time of these procedures. Ghazanfar et al. had a similar finding: a reduced incidence of RGC/DGE in patients who started clear liquids 24 hours before compared to patients who were on a regular diet [15].

Our study had its own significant limitations as far as looking at the prevalence of CSDGE related to GLP-1 analogs. We had only 34 patients confirmed to be on GLP-1 RA at the time of their upper endoscopy. Also, cases of patients with CSDGE were not matched with controls not using GLP-1 RA.

Considering these results, some obvious questions still need to be answered about the quality of the studies currently linking GLP-1 RA with DGE or gastroparesis. While there may very well be a relevant association albeit small, most studies reviewed in the cause of this study and even some referenced by ASA have not been well controlled for confounders. This leaves the question of whether simply holding GLP-1 RA will limit the incidence of RGC or CSDGE in patients undergoing elective endoscopies and, even more importantly, reduce their risk of aspiration. Another vital question begging for an answer is whether we are willing and able to hold all medications linked with DGE or gastroparesis as well as eliminate all reversible conditions linked with it. This does not seem to have been done in a rigorous fashion in most published studies. 

Irrespective of the position taken, an even more critical question is how and what can be done to reduce the risk of aspiration among patients undergoing endoscopies. The obvious answer is to assess this risk on a case-by-case basis, as already proposed by the AGA. It appears that a guideline recommending withholding these medications in all patients using GLP-1 analogs was done in haste. Without a well-designed controlled trial comparing the incidence of DGE on EGD in patients on GLP-1 RA between patients on GLP-1 RA and in those who had medications held for at least seven days prior, with a control population of diabetics and/or obese patients who are not on such medications. Such Studies may be able to focus on the small minority of patients with clinically significant RGC/DGE caused by GLP-1 RA and help maintain patient safety while avoiding withholding these medications on all patients per current guidelines.

## Conclusions

In conclusion, in an unmatched retrospective analysis of patients undergoing EGD, we did not find a significant contribution of GLP1 agonists to the problem of clinically significant RGC/DGE among asymptomatic patients. The contribution of GLP-1 RA in causing clinically significant RGC/DGE in those having an EGD and colonoscopy during the same session was even smaller. At this time, we feel that GLP-1 RA should be held for a week prior to EGD in patients with symptoms of bloating/fullness and nausea, but we do not have a compelling reason to hold the medication in those who are asymptomatic. We should await more robust and well-designed studies that will address limitations in current evidence before such a blanket recommendation is made.
